# Supplementary material for: Universal Health Coverage and the Pacific Islands: An Overview of Senior Leaders’ Discussions, Challenges, Priorities and Solutions, 2015–2020
Source: Int J Environ Res Public Health. 2022 Mar 30;19(7):4108. doi: 10.3390/ijerph19074108 (PMC8998582; doi:10.3390/ijerph19074108)
Supplement: Supplementary file 1 [file ijerph-19-04108-s001.zip › Supplementary File S1.pdf]

Supplementary file S1. Pacific Island Countries and Territories

American Samoa

Cook Islands

Federated States of Micronesia

Fiji

French Polynesia

Guam

Kiribati

Marshall Islands

Nauru

New Caledonia

Niue

Commonwealth of the Northern Mariana Islands

Palau

Papua New Guinea

Pitcairn Island

Samoa

Solomon Islands

Tokelau

Tonga

Tuvalu

Vanuatu

Wallis and Futuna
