# Supplementary material for: Universal Health Coverage and the Pacific Islands: An Overview of Senior Leaders’ Discussions, Challenges, Priorities and Solutions, 2015–2020
Source: Int J Environ Res Public Health. 2022 Mar 30;19(7):4108. doi: 10.3390/ijerph19074108 (PMC8998582; doi:10.3390/ijerph19074108)
Supplement: Supplementary file 1 [file ijerph-19-04108-s001.zip › Supplementary File S2.pdf]

Supplementary File S2. Literature search strategy (OVID)

1. universal health insurance/ OR ("universal health" OR UHC).mp
2. pacific islands/ OR exp melanesia/ OR exp micronesia/ OR exp polynesia/
3. ("american samoa" OR "cook islands" OR ("federated states of micronesia" OR Micronesia OR FSM OR yap OR chuuk OR kosrae OR phonpei) OR fiji OR ("french polynesia" OR "polynesie francaise") OR guam OR kiribati OR ("marshall islands" OR RMI) OR nauru OR ("new caledonia" OR "novelle caledonie") OR niue OR ("northern mariana islands" OR CNMI) OR palau OR ("papua new guinea" or PNG) OR pitcairn OR (samoa OR "samoan islands") OR "solomon islands" OR tokelau OR tonga OR tuvalu OR vanuatu OR ("wallis and futuna" or "wallis et fatuna")).mp.
4. 2 and 3
5. 1 and 4
